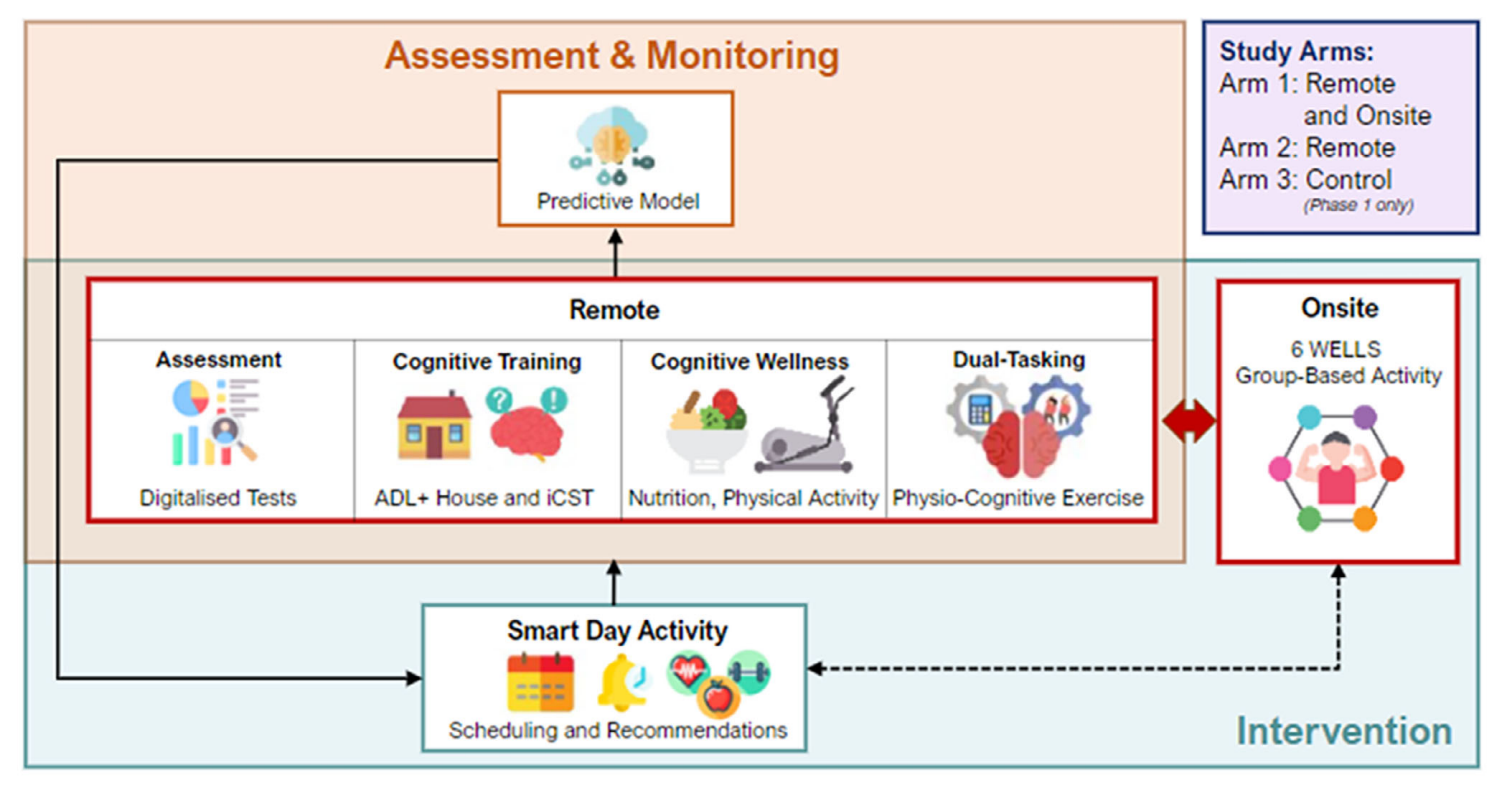# ADL+ 2.0 AI‐assisted multidomain digital intervention for prevention of cognitive decline: Protocol for an implementation research study in Singapore

**DOI:** 10.1002/alz70860_105383

**Published:** 2025-12-23

**Authors:** Wee Shiong Lim, Audrey Yeo, Kalene Pek, Sabrina Lau, Jia Qian Chia, Justin Chew, Ezra Ho, Ze Ling Nai, Woan Shin Tan, Yew Yoong Ding, Jun Pei Lim

**Affiliations:** ^1^ Institute of Geriatrics and Active Ageing, Tan Tock Seng Hospital, Singapore, Singapore; ^2^ Lee Kong Chian School of Medicine, Nanyang Technological University, Singapore, Singapore; ^3^ Geriatric Education & Research Institute, Singapore, Singapore

## Abstract

**Background:**

Despite strong evidence linking lifestyle modifications to reduced dementia risk, uptake of community preventative strategies remains low. Although digital health interventions offer scalable solutions, their implementation amongst older persons in real‐world settings is underexplored, including what works (blended vs digital only) and how, why and for whom it works (context, barriers and facilitators to implementation). We aim to describe the Phase 1 (test‐bedding) protocol for a community‐based theory‐guided AI‐assisted multidomain digital intervention (ADL+ 2.0) using implementation science approaches.

**Methods:**

Comprising remote (app‐based assessment and intervention with smart scheduling and recommendations) and onsite (6‐WELLS group‐based weekly activity) components, ADL+ 2.0 incorporates behavioural theories such as gamification and Self‐Determination Theory. The 6‐month intervention will be introduced at 12 Active Ageing Centres (AACs) across Singapore targeting older persons without dementia who are at risk of cognitive decline. Using a Type 1 hybrid implementation‐effectiveness study design, we will employ a cluster randomised trial design to compare effectiveness and cost‐effectiveness between the three groups (remote and onsite; remote only; and control) and to evaluate outcomes and determinants of implementation using the Consolidated Framework for Implementation Research (CFIR) with Outcomes Addendum (2022).

**Results:**

Effectiveness will be assessed via cognitive, functional, social and quality of life outcomes. The primary outcome will be change in composite and individual domain z‐scores via standardized neuropsychological test battery measured at 3‐monthly intervals up to 9 months (3‐month post‐intervention). Using a mixed methods approach, qualitative data about determinants to implementation (context, facilitators and barriers) derived from interviews and focus group discussions, will be mapped onto implementation strategies from the Expert Recommendations for Implementing Change (ERIC) to inform Phase 2 (scale‐up). Quantitative assessment of key implementation outcomes (acceptability, appropriateness, and feasibility) across clusters will also be collected.

**Conclusion:**

We describe the implementation research protocol for a population‐level, theory‐guided, AI‐assisted digital intervention that overcomes limitations in prior multidomain intervention studies to prevent cognitive decline. Insights from Phase 1 (test‐bedding) will inform the development and deployment of relevant, feasible and effective implementation strategies in phase 2 (scale‐up) to other AACs and new community implementation partners such as residential zones and primary care referrals.